# A220 MORTALITY IN PATIENTS WITH ULCERATIVE COLITIS UNDERGOING COLECTOMY (2016-2020)

**DOI:** 10.1093/jcag/gwad061.220

**Published:** 2024-02-14

**Authors:** N S Ahmed, S Krawchuk, k buhler, I Stukalin, J Besney, A Shaheen, C Seow, K Novak, R Ingram, C Lu, G G Kaplan, R Panaccione, C Ma

**Affiliations:** Internal Medicine, University of Calgary, Calgary, AB, Canada; University of Toronto, Toronto, ON, Canada; Internal Medicine, University of Calgary, Calgary, AB, Canada; Gastroenterology, University of Calgary, Calgary, AB, Canada; Internal Medicine, University of Calgary, Calgary, AB, Canada; Internal Medicine, University of Calgary, Calgary, AB, Canada; Internal Medicine, University of Calgary, Calgary, AB, Canada; Internal Medicine, University of Calgary, Calgary, AB, Canada; Internal Medicine, University of Calgary, Calgary, AB, Canada; Internal Medicine, University of Calgary, Calgary, AB, Canada; Internal Medicine, University of Calgary, Calgary, AB, Canada; Internal Medicine, University of Calgary, Calgary, AB, Canada; Internal Medicine, University of Calgary, Calgary, AB, Canada

## Abstract

**Background:**

Despite an expanding armamentarium of medical treatment options, the 10-year cumulative risk of colectomy for patients with ulcerative colitis (UC) remains 5-10%. Surgery for UC is associated with a substantial burden of mortality. A previous meta-analysis of population-based studies found that postoperative mortality was 0.7% of patients undergoing elective surgery and 5.3% of patients undergoing emergent colectomy.

**Aims:**

Given improvements in managing acutely ill patients with UC, we aimed to evaluate contemporary rates of postoperative mortality following colectomy.

**Methods:**

We analyzed data in the National Inpatient Sample (NIS) for 2016-2020. The NIS is an all-payer administrative health database, capturing information from ampersand:003E7 million inpatient admissions at ampersand:003E1000 hospitals across the United States annually. All analyses were weighted to account for the complex stratified survey design. Adult patients (≥18 yrs) with a primary diagnosis of UC undergoing colectomy were identified with ICD-10 coding. Rates of in-hospital postoperative mortality were calculated, and predictors of mortality were evaluated in survey-adjusted logistic regression.

**Results:**

A total of 8570 hospitalizations for patients with UC undergoing colectomy were included. Mean age at colectomy was 44.5 years and 47% of patients were female. Emergency colectomy was performed in 38.2% [95% CI: 35.9%, 40.7%] of patients, and was attempted laparoscopically in 55.9% [53.1%, 58.7%]. Overall mortality from 2016-2020 was 1.2% [0.8%, 1.9%], but was 0.2% [0.1%, 0.8%] for elective surgery and 2.9% [1.9%, 4.5%] for emergent surgery. Stratified rates of mortality are summarized in **Table 1**. In multivariable analysis, age was not an independent predictor of mortality but laparoscopic surgery (adjusted odds ratio 0.24 [0.06-0.98], p=0.047) and elective resection (aOR 0.16 [0.04-0.68], p=0.01) were associated with a lower risk of postoperative death.

**Conclusions:**

Approximately 1 in 100 patients undergoing colectomy for UC will die postoperatively. This risk is highest in comorbid patients undergoing open laparotomy or emergency colectomy. The risk of mortality in both emergent and elective settings is lower than previously reported.

Table 1. Stratified risks of mortality after colectomy for ulcerative colitis

**Funding Agencies:**

None

**Table:** 

Characteristic		Postoperative Mortality (95% CI)	p-value
Sex	Male Female	1.2% [0.7%, 2.2%] 1.3% [0.7%, 2.3%]	0.94
Race	White Black Hispanic	1.2% [0.7%, 1.9%] 2.9% [0.7%, 11.0%] 0.8% [0.1%, 5.4%]	0.11
Primary Payment Method	Medicare Medicaid Private insurance	4.2% [2.4%, 7.1%] 1.6% [0.5%, 5.0%] 0.5% [0.2%, 1.1%]	ampersand:003C0.001
Elixhauser Comorbidity Index	0 1 ≥2	0.0% 0.2% [0.0%, 1.6%] 2.3% [1.5%, 3.5%]	ampersand:003C0.001
Median household income for ZIP code	0-25th percentile 25-50th percentile 50-75th percentile 75-100th percentile	1.3% [0.5%, 3.4%] 1.2% [0.5%, 2.9%] 1.1% [0.5%, 2.6%] 1.2% [0.8%, 1.9%]	0.98
Surgery type	Open Laparoscopic	2.4% [1.5%, 3.8%] 0.3% [0.1%, 1.9%]	ampersand:003C0.001
Surgical urgency	Elective Emergent	0.2% [0.1%, 0.8%] 2.9% [1.9%, 4.5%]	ampersand:003C0.001
Hospital size	Small Medium Large	1.3% [0.3%, 5.2%] 2.0% [0.1%, 4.1%] 0.1% [0.6%, 1.8%]	0.32
Hospital region	Northeast Midwest South West	1.1% [0.4%, 2.9%] 1.3% [0.6%, 2.8%] 1.2% [0.5%, 2.5%] 1.3% [0.5%, 3.5%]	0.99
Hospital type	Rural Urban non-teaching Urban teaching	0.0% 2.0% [0.5%, 7.5%] 1.2% [0.8%, 1.9%]	0.71

